# Exploring safety in disaster-induced displacement relocation site schools in Tokwe-Mukosi, Zimbabwe

**DOI:** 10.4102/jamba.v13i1.842

**Published:** 2021-08-13

**Authors:** Munyaradzi Chidarikire, Dipane Hlalele, Kudzayi S. Tarisayi

**Affiliations:** 1 School of Education, University of KwaZulu-Natal, Pinetown, South Africa; 2 Department of Curriculum Studies, Stellenbosch University, Stellenbosch, South Africa

**Keywords:** safe schools, photovoice, disaster-induced learning ecologies, displacement, learners, Zimbabwe

## Abstract

The victims of the Tokwe-Mukosi disaster-induced displacements in Zimbabwe were relocated to Chingwizi, Chisase and Masangula. This qualitative article explores the safety of learners in Tokwe-Mukosi disaster-induced displacement schools. The safety of learners in schools influences the education’s agenda. Most of the studies on the safety of learners focus on rural and urban schools at the expense of schools located in disaster-induced displacement sites. This article seeks to explore learners’ safety in disaster-induced displacement schools in Zimbabwe. The 2013 Zimbabwe Constitution and *the Ministry of Primary and Secondary Education Act 1987* (Section 25:04) emphasise the importance of learners and teachers’ safety. The critical emancipatory research (CER) was used as a theoretical framework in this article. The participants responded to the research question: *Are school authorities ensuring safety in disaster-induced displacement relocation site schools, Tokwe Mukosi*? Data were generated through photovoice. Participatory action research was used as a research methodology with 15 participants being purposively selected. This article found that learners were not safe in the schools located in disaster-induced displacement areas in Tokwe-Mukosi. To mitigate safety problems, participants recommended that the government and other stakeholders should collectively build a safe learning environment.

## Introduction and contextualisation of the problem

This article seeks to explore the safety of learners in disaster-induced displacement relocation site schools in the Tokwe-Mukosi area in Zimbabwe. There is dearth of literature on the safety of learners in disaster-induced displacement relocation site schools in Zimbabwe. However, there is growing literature on the Tokwe-Mukosi floods and the subsequent displacements. In 2014 flooding of the Tokwe-Mukosi basin led the government of Zimbabwe to relocate people to Chingwizi, Chisase and Masungula. Tarisayi (2014) established that the people who were relocated to Chingwizi lost their livelihoods because of the disaster-induced displacements. Hove (2016) revealed that the displaced people became victims of poverty and marginalisation. Tarisayi (2018) opined that the missing link in the Tokwe-Mukosi disaster-induced displacement was the traditional leadership. Chendume (2016) concluded that there were human rights abuses in the relocation of people displaced by the Tokwe-Mukosi floods. From the given studies, it emerges that there is very little known about the safety of learners and teachers in the Tokwe-Mukosi schools established for victims of disaster-induced displacements in Zimbabwe.

Therefore, this article intends to contribute knowledge in relation to the role of school authorities in solving safety problems in disadvantaged disaster-induced displacement areas and thereby increasing the safety and effectiveness of education in schools.

## Theoretical framing: Critical emancipatory research

The critical emancipatory research (CER) was used as a theoretical framework in this article. Vinz (2016:1) defined theoretical framework as ‘a good, strong scientific research base that provides support for the rest of your thesis’. We used CER as a theoretical framework because it advocates for equality, justice and empowerment of local people in identifying their problems and finding solutions. This is supported by Noel (2016), who asserted that:

Emancipatory research is a research perspective of producing knowledge that can be of benefit to people who may be marginalised for reasons of race, gender, sexual orientation, disability, and economic background. (p. 1)

The Tokwe-Mukosi communities in disaster-induced displacement sites are disadvantaged and their views were not considered in previous research studies (Chidarikire 2017). However, in this article we succeeded in engaging the participants actively and factored in their views. In addition, Etonge (2014) held that the:

Choice of CER is inevitable for such a paper, since it proposes the solving of a problem by using values such as democracy, social justice, sustainable livelihood, empowerment and emancipation. (p. 31)

Accordingly, CER supports and promotes the total engagement of local communities and enhances ownership of safety strategies at their schools and the mobilisation of resources to implement safety measures such as building safe classrooms. Similarly, Nkoane (2012) explained that the:

Researcher and research participants work together to develop understanding and knowledge about the nature and root cause of an undesirable situation, in order to design strategies and marshal support to effect change. (p. 98)

However, in most schools in Zimbabwe, Zvirevo (2013:46) found that ‘learners, poor community members, and teachers are not consulted when programmes are formulated in Zimbabwe’. Therefore, the involvement of community members from the disaster-induced displacement areas in this article created a platform for them to actively provide solutions to school safety problems that hinder educational processes. Fournier et al. (2007) elaborated that:

CER stresses the importance of people speaking from their own experience, identifying a common theme among their individual situations, creating an analytical perspective from which to relate their situation to root causes, developing solutions and strategies for change. (p. 4)

As a result, CER gives opportunities for participants in disaster-induced displacement areas to air their views in formulating a culturally relevant and all-education peer-counselling strategy. We hold that CER is empowering and it changes the lives of disadvantaged community members. The researchers in this article argue that CER allows for the transformation and empowerment of participants, which is the purpose of this article.

## Literature review

### The legal policies that deal with the safety of learners

The Ministry of Primary and Secondary Education’s (2019) vision statement begins with: ‘to be the leading provider of quality education’. As the provision of quality education is centred on safety principles and actions concerning learners, an analysis of the Government of Zimbabwe (2013) and *the Ministry of Primary and Secondary Education Act* (25:04) were imperative, to understand the issues of safety in schools from the policy and legal framework angles. The Constitution, Section 81, stipulates that ‘every child should be protected from harm’. Some of the rights of learners included in the constitution include the right to free primary education and affordable secondary education, the right to appropriate shelter and the right of freedom from punishment and child labour practices (learners). This shows how Zimbabwe respects the rights of the learners as enshrined in the supreme law of the country – and this should be strictly adhered to (Human Rights Watch Report 2015; Machingura 2018). No person or any obstacle within and out of school premises should cause pain, harm and suffering to learners in Zimbabwe. In addition, Zimbabwe is a signatory to national and international conventions that enforce the upholding of the rights of learners – these are the *Universal Declaration of Human Rights*, *International Covenant on Economic, Social and Cultural Rights*, the *Convention on the Rights of the Child* and the *African Charter on the Rights and Welfare of the Child*. The constitutional obligations were factored into *the Zimbabwe Ministry of Primary and Secondary Education Act* (Section 25:04) where the role of teachers, parents and school authorities should include safe learning environments for effective teaching and learning processes.

Accordingly, constitutional obligations and directives from educational policies regarding school safety are explicitly stated. Teachers, parents and responsible authorities are expected to provide educational facilities and safe learning environments, free from verbal, physical and sexual abuses. Furthermore, role-players are expected to identify and report any sign of abuse and injury (Muridzo, Chikadzi & Kaseke 2018). Also, both the Constitution and the *Education Act* allow school authorities, parents and Non-Governmental Organisations (NGOs) to provide food for the learners to combat malnutrition as denying learners food is one form of abuse (Tshili 2017). It is imperative for teachers and school authorities to understand educational policies and constitutional law concerning learners and school safety; however, Mutale (2015:35) stated that ‘most parents lack access to state policies and regulations’. However, there are safety concerns in Tokwe-Mukosi disaster-induced displacement schools, and this motivated the research presented in this article.

### Unsafe environment in schools

Effective education takes place in safe teaching and learning environments. Unsafe buildings such as pit toilets affect the successful implementation of educational processes. In South Africa, two learners died at the pit toilets in their schools in 2014 and 2018 (BBC News 2018; Lekalakala 2018; Marchesi 2018). Some studies carried out in other provinces in Zimbabwe (different from the area where this study was conducted) found that most school learning spaces are against education (Chitiyo & Wheeler 2004; Foundation for Human Rights Initiative 2013; Hlatywayo & Muranda 2014; Ncube 2014). In Botswana, the death of learners on school premises causes learners to avoid going to the toilet because of fear caused by such deaths (Donohue & Bornman 2014; Gina 2017). In addition, pit toilets in schools in Nigeria cause the spread of diseases such that many students drop out from school because of sicknesses thereof (Ago et al. 2012; Amoram 2017; Kolo et al. 2017; Yasuf 2018). The causes of sicknesses emanate from the fact that most toilets in schools are not regularly cleaned because of the lack of cleaning detergents, toilet paper and water to wash their hands after using the toilets (Lekalakala 2018; Louton et al. 2015; Matthews 2018). In Malawi, Holm (2018:3) found that ‘no school had facilities that fully met the needs of students with disabilities - not even private schools which are often thought to provide better services’. In relation to inaccessibility of toilets by teachers and learners with disabilities in Zimbabwe, Hlatywayo and Muranda (2014:127) found that the lack of ‘toilet facilities was cited as one of the main reasons for the high dropout rate among learners with disabilities’. Apart from sanitation challenges, learners are endangered when they prefer to learn under trees rather than take the risk of being in a dilapidated classrooms, which may collapse at any moment – but there are other dangers, when learning under trees, such as harsh inclement weather (Gutuza 2015; Mpindu 2012; Ndlovu 2013; Nyandoro et al. 2013). Hence, there is a need for the government and school authorities in Tokwe-Mukosi’s disaster-induced displacement relocation site learning ecologies to provide educational facilities that are conducive to quality teaching and learning.

### School authorities as drivers of safety in schools

One of the duties of school authorities in learning ecologies is to provide safety for their learners (Constitution of Zimbabwe 2013; Nyandoro et al. 2013; Sango 2016). One of the ways to promote safety for the learners is to conscientise all role-players on the importance of safety at learning institutions (Motoko 2017; Nconsta & Shumba 2013). This will activate the community members and others including NGOs to mobilise resources to build safe classrooms and play-centres (Masuku 2018; Ncube 2016; Tshabalala 2015). In addition, school teachers in schools should also supervise learners in classrooms, in play-centres and when they visit toilets and other school buildings (Bloom 2018; Dewure Secondary School Newsletter 2019). There are studies that found that the lack of supervision contributes to unsafe learning environments (Chireshe 2013; Mapfumo 2014; Mapuranga & Nyakudzuka 2014; Ngwenya & Pretorius 2014). Furthermore, teachers and community members should teach learners about how to live safely, for example not to walk alone in the bush or other remote spots. Recently, in one rural area in Zimbabwe, far away from the place where this study was conducted, one girl was raped in the bush after being waylaid as she was walking home (Mzwazi 2018). Furthermore, teachers and community members should not verbally, sexually, physically and emotionally abuse learners, as there are many media reports and literature studies that found evidence of adults abusing learners at schools (Gudyanga 2014; Laiton 2017; UNICEF Country Report 2019; Vambe 2018; Zhou 2015). The role of a teacher is to act as a parent of the learner (*in loco parentis*), and the role of a parent is to provide love, care and empathy to learners (*Education Act 1987*). School heads should also protect teachers from being violated through verbal and physical abuse by learners, other teachers, school heads and parents. One learner murdered his teacher because he accused him for making him (the learner) to fail (Mashaba 2018; Michaels 2018). Also, there are community members who violently attack teachers and learners verbally or physically causing emotional, psychological and physical pain (Langa 2015; Zengeya 2016; Zondi 2017). There is a dearth of literature on the role of school authorities on the safety issues in schools located in disaster-induced displacement schools. This has necessitated the study of this article.

## Methodology: Participatory action research

Participatory action research (PAR) was utilised in this article to actively engage learners, teachers and school authorities in the discourse of exploring safety in disaster-induced displacement relocation site schools in Tokwe-Mukosi, Zimbabwe. Participatory action research methodology is empowering in nature, as explained by Dube (2016:8) that ‘the strength of PAR lies in the fact that it recognises the capacity of participants in contributing to the research process towards improvements and social transformation’. Participants were actively engaged in all stages of research. This is contrary to other studies that involve only the participants in data generation. In addition, PAR according to Nkoane (2015:12) is used ‘for its transformative endeavours and emancipatory consciousness’.

The participants were able to identify safety problems, the causes of safety problems, ways to mitigate these challenges and their roles in advancing safety of learners in their schools (Zuber-Skerrit 2015). We hold that PAR allows the marginalised community members from the disaster-induced displacement areas to identify their societal problems such as violence, sexual abuse, inadequate educational facilities and unsafe learning environments and thus provides solutions to overcome these problems.

Furthermore, PAR enables learners to use photovoice, the data generation method that was used in this article (Evans-Agnew & Rosemberg 2016). Photovoice technique is empowering in nature, as it allows people from the disadvantaged communities to use technological gadgets they have not used. Therefore, there is a connection between PAR and the data generation technique – photovoice, in that PAR has an empowering agenda (Dube 2020). In addition, PAR resonates with this article’s theoretical framework CER because it advocates for the empowerment and emancipation of marginalised research participants (Hlalele 2018). In this article, therefore, participants used photovoices to express school safety/non-safety concerns through pictures. Photovoice helped to record, reflect and promote dialogues concerning safety amongst disaster-induced displacement relocation site schools in Zimbabwe. The participants were put into two focus groups, each focus group had seven and eight participants. Johnson and Christenen (2007) aver that:

A focus group is a type of a group interview in which a moderator leads a discussion with a small group of individuals to examine in detail, how the group members think and feel about a topic. (p. 185)

According to Creswell and Poth (2018) focus group should be small to allow members to actively participate in the study. Ground rules were agreed upon by members in each focus group. Each focus group had a secretary to record the group discussions. Group discussions were helped for 1 h and 30 min for 3 days. The 15 participants were selected through purposive sampling method. Purposive sampling technique allowed us to choose knowledge-rich participants, who have expertise in safety issues in schools located in disaster-induced displacement relocation site schools (Creswell & Poth 2018).

## Data generation and data analysis

This qualitative article used the photovoice technique to generate data. Photovoice is defined by Ebrahimpour, Esmaeilie and Varaei (2019:216) as an ‘innovative data collection method which can provide deep and rich information for researchers; it is a term consisting of two words, photo and voice’. It was first used by Wang and Buriss as a research methodology in 1997 in conjunction with community-based PAR (Evans-Agnew & Rosemberg 2016). The proponents of photovoice, Wang and Buriss (1997), explained that research participants used cameras to capture community issues thus empowering them to be active participants in research processes to create viable solutions for their communities. The participants took photos by using the camera the researchers gave them. Also, two participants downloaded photos from the Internet by using the researcher’s phone; it was their first time to use phones in this regard. This supports our view that a photovoice technique is empowering (Evans-Agnew & Rosemberg 2016). Photovoice allowed participants to articulate their views by using pictures they took from their schools in response to the research: *Are school authorities ensuring safety in disaster-induced displacement relocation site schools, Tokwe-Mukosi*? The participants discussed the pictures and were giving views on the dangers imposed on learners in disaster-induced learning environments and solutions to mitigate these challenges.

We used focus group discussions as an instrument to generate data from the participants. The data generated from participants were categorised into themes. Braun and Clarke (2006:10) argue that, ‘theme captures something important about data in relation to the research question, and presents some level of patterned response or meaning with the data set’. The generated data were divided into two themes: Theme 1: The challenges faced by safety in disaster-induced displacement schools and Theme 2: Solutions to mitigate challenges faced by safety issues in disaster-induced displacement schools. Trustworthiness was enhanced by a pilot study, which eliminated ambiguities in data generation instrument. To enhance trustworthiness of data, the researchers used member checking of the data from focus group discussions. To have access to the participants, we obtained the relevant clearance as part of advance protocol to conduct this study in the selected secondary schools from the Ministry of Primary and Secondary Education. The participants signed informed consent forms. Data generation commenced with giving assurance to participants that their responses were to be treated with the strictest confidentiality. In maintaining privacy, each participant’s name was saved by using an anonymous name. In addition, participants’ parents and guardians were informed about the purpose of the study in a bid to obtain their consent concerning the participation of their children in the study and in turn we requested them to assent to their children’s involvement in data generation.

## Discussion and findings

The discussion and findings are derived from the pictures taken by participants and from other pictures, which were downloaded from the Internet sources by participants. This was in line with the concept of empowering the marginalised community members’ photovoice. The data were divided into two themes: challenges and solutions of safety in disaster-induced school sites.

### Theme 1: Challenges on the roles of school development committees towards the safety of learners in disaster-induced displacement schools

This section focusses on the roles of school development committees towards the safety of learners in disaster-induced displacement schools. The participants argued that many school development committee members in schools located in disaster-induced displacement sites have limited knowledge of their roles in promoting safety in schools.

Participant 2 explained that:

‘I was deployed to be the head of this school (Shumba [psyndonym used to protect participant’s identity]). I realised that most school development community members were not aware of the importance of safety at the school. They did not mend broken windows and did not clean dirty classroom walls. They were never informed by the previous school head about how they should assist the school and were not aware that it is their responsibility as stipulated in *the Education Act 1987*.’ (P2, male, primary school head, 25 March 2019)

**FIGURE 1:**
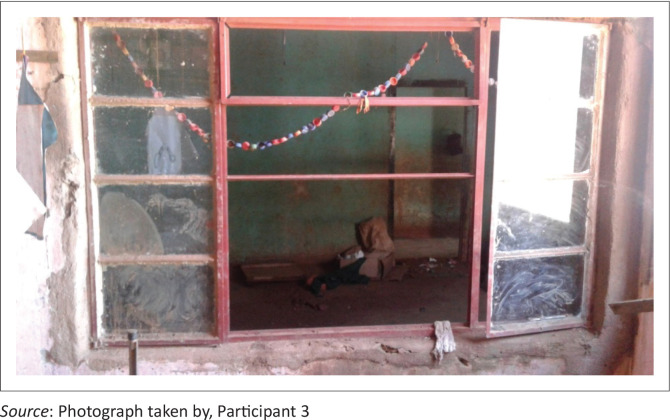
Classroom with broken window panes, exposing learners to bad weather and thieves.

More so, school development committees in these schools are not aware that they should mobilise money to repair schools’ equipment:

Participant 3 commented that:

‘I have taken a picture [*Figure 1*] which shows the broken windows of one of our classrooms. These windows were broken 3 years ago. They have not been repaired. The school development committee has not mobilised money to buy new window panes because they thought the government is responsible for repairing schools’ equipment.’ (P3, female, school teacher, 25 March 2019)

From the given responses based on the picture and verbatim shown here, there are some school development committees in disaster-induced displacement relocation site schools, which are not knowledgeable of their roles in relation to the provision of safety at their schools. Most school development committees in rural areas have no skills and expertise in school safety issues; this is shown in Gutuza (2015), Mapolisa (2014) and Mapuranga and Nyakudziwa (2014). The picture (Figure 1) shows the broken windows that expose learners and teachers to bad weather. We observed that some learners at one disaster-induced displacement school had flue because of dust that entered through broken windows. A closer look at the picture shows rubbish inside the classroom and this exposes learners to disease, thus classrooms should be clean. Therefore, safety of learners and teachers is compromised.

The next section describes the learning spaces in disaster-induced displacement relocation site schools as unsafe to both teachers and learners in Zimbabwe. The classrooms in such schools are not safe zones. The majority of learners in such schools are placed under trees, semi-built classrooms and open spaces.

**FIGURE 2:**
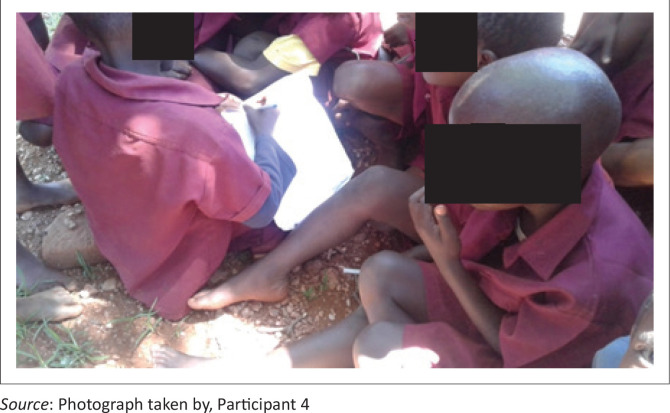
Learners learning sitting on the school grounds.

Participant 4 stated that:

‘This picture [*Figure 2*] is from my school. Learners are learning under the tree because there are inadequate classrooms. It is difficult to learn and teach outside if it is hot, windy, cold and rainy. Some learners and teachers get sick because of dusty and unfavourable weather.’ (P4, male, school development committee member, 25 March 2019)

The picture (Figure 2) shows one semi-built classroom at one school in a disaster-induced displacement relocation site school.

**FIGURE 3:**
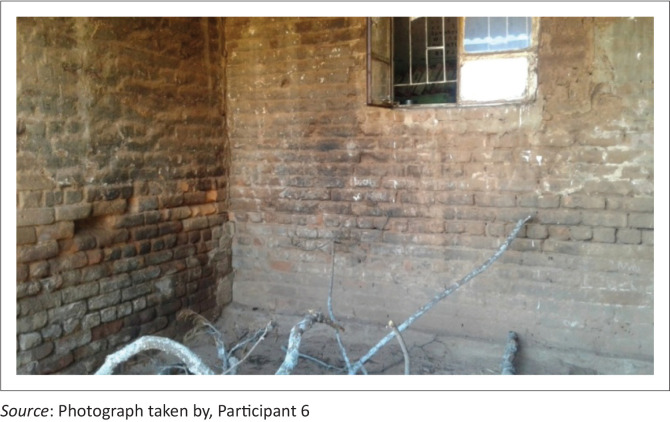
Learning classroom with firewood inside, exposing learners to scorpions.

Participant 6 highlighted that:

‘This is my nearby school. Classroom buildings are semi-built and dilapidated, it has dirty walls, a no roof and cracked walls. These pose dangers to learners and teachers. Fire wood are kept at the corner of the classroom, one learner was bitten by snake that was hiding in the wood.’ (P6, female, leader of local NGO advocating for children rights, 25 March 2019 [*see Figure 3*])

In addition, the toilets in schools located in disaster-induced places expose learners to danger.

**FIGURE 4:**
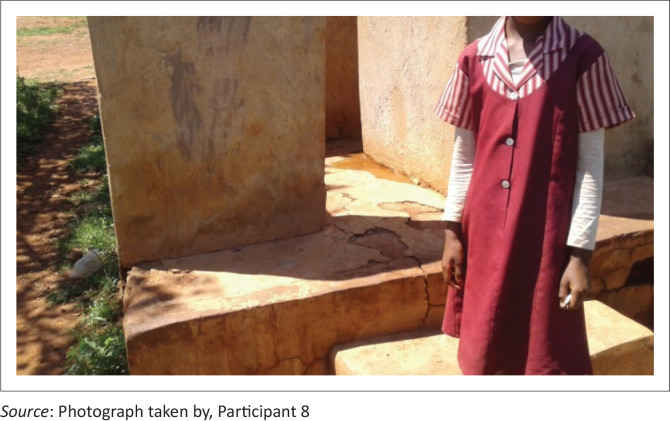
Learner near school toilet with cracked walls and floors.

Participant 8 observed that:

‘This toilet has broken doors, poor ventilation, is dirty and small in size, and has no roof tops. Learners with wheel chairs cannot access these toilets. Toilet seats are very high such that learners in lower grades cannot access them. One learner fell into the pit toilet and was badly injured, and also they are not user-friendly to people with disabilities. I am using walking sticks. I struggle to enter my classroom because there are steps at the door and the floor is slippery. One of my learners who was using a wheelchair took a transfer to a school in a town, she could not access school buildings such as toilets using her wheelchair. Most of our learners come to school without shoes and go to dirty toilets, thereby being exposed to diseases.’ (P8, male, local ward councilor, 25 March 2019 [*see Figure 4*])

From the given pictures, participants argued that many school buildings are not safe for learning and teaching to take place. Some schools use open spaces as learning venues, some classrooms are incomplete and the other buildings are in a dilapidated state and toilets have high steps that make young learners to fall and injure themselves (Birch & Wachter 2011; Marchesi 2018; Mutale 2015). Rooms have narrow doors and steps that make learners with disabilities to struggle to access using wheel chairs, some fell down and get injured. We observed that most learners and teachers are exposed to learning environments that are prone to health hazards (Foundation for Human Rights Initiative 2013:^3^). Therefore, the government, parents and other organisations should step up their efforts and mobilise resources to build new blocks, renovate buildings and redesign classrooms and office blocks to make them user-friendly to people with physical challenges.

The next section deals with the issue of corporal punishment in schools.

Corporal punishment in schools makes schools unsafe. Some learners are subjected to corporal punishment in schools located in disaster-induced displacement relocation site schools.

Participant 5 said:

‘Corporal punishment is only allowed to be implemented by the school head or a delegated member of the staff. Not all teachers are allowed by law to beat up learners. However, the reality in the classroom is that teachers are beating up learners who misbehave. This creates a phobia among learners and they end up feeling unsafe.’ (P5, female, school inspector, 25 March 2019)

In support of the dangers of corporal punishment, Participant 6 explained:

‘One learner at our school died after the teacher beat his head. After this incident 23 learners transferred from our school. Corporal punishment is dangerous and attacks the safety of learners.’ (P6, female, leader of local NGO advocating for children rights, 25 March 2019)

On the contrary, there are other participants that support corporal punishment, Participant 7 argued:

‘Corporal punishment is vital in instilling discipline amongst learners. These days learners are disobeying teachers and parents because corporal punishment is no longer in use.’ (P7, male, police office, 25 March 2019)

**FIGURE 5:**
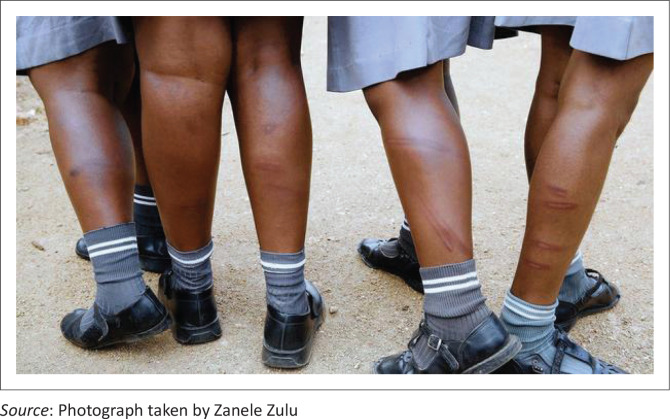
Learners’ injured legs after being beaten by their school teacher.

The given picture (Figure 5) and participants’ views show. Most of the injury scars on the legs will be permanent. The learners feel unsafe at school because of the use of corporal punishment (Chinowaita 2015; Diraditsile 2018; Dube & Hlalele 2018; Laccino 2014; Mthethwa 2018; Vinga 2014). However, some participants are of the view that corporal punishment brings discipline to learning institutions (Vambe 2018; Zhou 2017), whilst some argued that it brings fear and pain in learners and there are teachers who have been fired, suspended and imprisoned after they injured learners whilst punishing them (Musarurwa 2017; Rupapa 2015; Zengeya 2016; Zondi 2017). As a result of the inhumane treatment caused by people who administer corporal punishment, the High Court judgement against corporal punishment was promulgated. Laiton (2017) stated that:

Judge Justice David Mangota has outlawed corporal punishment against learners at school or home by teachers and parents saying that the practice is a contravention of the country’s supreme laws. (p. 1)

We observed that most participants agreed that corporal punishment is rampant in disaster-induced displaced schools in Zimbabwe.

This article does show not only safety challenges faced by learners and teachers in disaster-induced displaced schools in Tokwe-Mukosi, but it also gives ways to enhance safety in these schools.

### Theme 2: Some of the solutions to mitigate challenges faced by safety issues in disaster-induced displaced schools

Following are ways to promote safety in disaster-induced displaced schools in Tokwe-Mukosi. This section focusses on the government and other stakeholders’ roles in building safe schools, and school development committees should be trained in safety issues.

Participant 8 said:

‘the government and non-governmental organisations should build adequate schools, other state-of-the-art education infrastructure and user-friendly toilets. This enhances learners and teachers’ safety in disaster-induced displaced schools in Zimbabwe.’ (P8, male, local ward councilor, 25 March 2019)

Participant 9 opined that:

‘Parents and learners’ relatives should contribute financially and materially to assist in building of schools and repairing of windows, doors and other aspects.’ (P9, female, educational psychologist, 25 March 2019)

On the contrary, school development committees should be trained on their roles and responsibilities in school safety.

Participant 10 is of the view that:

‘School development committees should be adequately trained in school safety issues. Lack of training incapacitates school development committees’ roles.’ (P10, male, school development committee member, 25 March 2019)

Participant 11 commented that:

‘Ministry of Primary and Secondary Education should hold training workshops and conferences to induct and train school development committees in disaster-induced communities.’ (P11, female, school head, 25 March 2019)

In addition, participants are of the view that teachers who implement corporal punishment on learners should be severely punished:

Participant 12 explained:

‘Teachers who beat learners should be severely punished by the Ministry of Primary and Secondary School and the courts. Courts should give deterrent sentences to teachers.’ (P12, male, police social worker, 25 March 2019)

Participant 13 said:

‘Teachers should be trained in the dangers of corporal punishment on learners. In addition, teachers should use other methods to deal with learners with unwanted-behaviour such as sweeping the class.’ (P13, female, social worker, 25 March 2019)

In promoting safety in schools, the participants are of the view that the government should assist disadvantaged schools in building safe schools. It is the responsibility of the government to build schools, medical facilities and other institutions if it relocates its people. Scholars such as Mzwazi (2018) argued that the government has constitutional responsibility to build schools in disadvantaged communities such as disaster-induced displacement site schools. In addition, the non-governmental organisations should also assist in building schools in marginalised societies. More so, the participants argued that the safety of learners and teachers can be enhanced if parents and relatives help in repairing the broken school windows and doors. School development committees should be adequately trained in school safety responsibilities. The Ministry of Primary and Secondary Education should periodically hold school development training workshops and conferences. Teachers should be trained on the dangers of corporal punishment, and deterrent sentences should be given to the teachers who execute corporal punishment on learners.

## Conclusion

From the data generated, the following conclusions were elicited relating to the safety concerns in many disaster-induced displacement relocation site schools in Zimbabwe. The majority of learning spaces in disaster-induced displaced schools are unsafe to both teachers and learners in Zimbabwe. Some of the school development committee members are not knowledgeable enough about the importance of safety at their schools; although some understand the significance of safety at their learning institutions, they lack the will and resources to implement safety measures. The government, non-governmental organisations and community members should adequately build safe schools in marginalised communities such as disaster-induced displaced communities. Most school development committees and teachers need training on safety issues and dangers of corporal punishment. Therefore, the Ministry of Primary and Secondary Education should train teachers and school development committees on the impact of unsafe learning environment on teaching and learning and how to deal with safety challenges in their schools.

## Recommendations

All disaster-induced displacement relocation site schools in Zimbabwe should implement strategies that alleviate harm and danger to teachers and learners. The following are some of the suggestions:

### Safety training workshops and awareness programmes

The training of teachers, learners, school heads and community members on safety measures and dangers of corporal punishment and bullying should be conducted regularly. Stakeholders who are rich in knowledge should help in creating and sustaining safe and conducive learning environments that enhance education in disaster-induced displacement schools. Sometimes learners, teachers and community members engage in unsafe behaviours because of the lack of understanding and knowledge.

### Role of school community members

School community members such as school development committee members, teachers, school heads, learners and other stakeholders should work together to create safe learning and teaching spaces. School authorities may partner with community organisations and the government to build, renovate school buildings and drill boreholes to provide safe drinking water. School community active partnerships help in the mobilisation of resources such as labour and building materials.

### School facilities should be user-friendly to people with physical challenges

The government of Zimbabwe through the Ministry of Primary and Secondary Education should make it mandatory that all school buildings should be user-friendly to learners and teachers with disabilities. Any school that is found to be breaching this should be censured.

### Stiff penalties on perpetrators of corporal punishment

The Ministry of Primary and Secondary Education should initiate awareness campaigns that effectively criminalise corporal punishment. Policies on banning the corporal punishment and other abuses should be regularly taught in schools and severe punishments should be given to perpetrators of these acts that make learning and teaching environment of schools unsafe.

### Counselling and treatment of the affected

Learners and teachers who are (were) physically and emotionally abusive should be counselled by professional counsellors, educational psychologists and other relevant health professionals. In addition, medical treatment should be provided to teachers and learners who have physical and other bodily pains. Counselling and treatment of the affected help them to develop better self-esteem and the ability to deal with inner and outer pain.
